# Running-Time Analysis of Brain Storm Optimization Based on Average Gain Model

**DOI:** 10.3390/biomimetics9020117

**Published:** 2024-02-15

**Authors:** Guizhen Mai, Fangqing Liu, Yinghan Hong, Dingrong Liu, Junpeng Su, Xiaowei Yang, Han Huang

**Affiliations:** 1 School of Physics and Electronic Engineering, Hanshan Normal University, Chaozhou 521041, China; 20200043@hstc.edu.cn; 2 School of Software Engineering, South China University of Technology, Guangzhou 510006, China; liudingrong@gzgs.edu.cn (D.L.); sujp.scut@outlook.com (J.S.); xwyang@scut.edu.cn (X.Y.); hhan@scut.edu.cn (H.H.)

**Keywords:** brain storm optimization (BSO), running time, average gain model, mutation operator, linear function

## Abstract

The brain storm optimization (BSO) algorithm has received increased attention in the field of evolutionary computation. While BSO has been applied in numerous industrial scenarios due to its effectiveness and accessibility, there are few theoretical analysis results about its running time. Running-time analysis can be conducted through the estimation of the upper bounds of the expected first hitting time to evaluate the efficiency of BSO. This study estimates the upper bounds of the expected first hitting time on six single individual BSO variants (BSOs with one individual) based on the average gain model. The theoretical analysis indicates the following results. (1) The time complexity of the six BSO variants is $O(\sqrt{n})$ in equal coefficient linear functions regardless of the presence or absence of the disrupting operator, where *n* is the number of the dimensions. Moreover, the coefficient of the upper bounds on the expected first hitting time shows that the single individual BSOs with the disrupting operator require fewer iterations to obtain the target solution than the single individual BSOs without the disrupting operator. (2) The upper bounds on the expected first hitting time of single individual BSOs with the standard normally distributed mutation operator are lower than those of BSOs with the uniformly distributed mutation operator. (3) The upper bounds on the expected first hitting time of single individual BSOs with the $U\left(-\frac{1}{2},\frac{1}{2}\right)$ mutation operator are approximately twice those of BSOs with the U(−1,1) mutation operator. The corresponding numerical results are also consistent with the theoretical analysis results.

## 1. Introduction

The swarm intelligence algorithm is one of the nature-inspired optimization algorithms that simulates the behavior of biological groups in nature [1,2,3]. Over the past two decades, many different types of swarm intelligence algorithms have been proposed, such as particle swarm optimization (PSO) [4,5], ant colony optimization (ACO) [6,7], artificial bee colony (ABC) [8,9], and brain storm optimization (BSO) [10,11,12]. Different from traditional methods, these algorithms solve problems by simulating the behavior of animal or human groups, with higher flexibility and adaptability.

BSO, a novel swarm intelligence algorithm, is inspired by the human brainstorming process. It is a continuous evolutionary algorithm that simulates the collective behavior of human beings. In recent years, BSOs have seen various practical applications in power systems [13,14,15,16], aviation design [17,18,19], mobile robot path planning [20,21], antenna design [22], financial optimization [23,24,25], and many other fields [26,27,28,29,30].

In addition, the theoretical analysis of BSO is also very important, especially for the practical application of BSO. The theoretical analysis benefits researchers in enabling them to understand the mechanism of the algorithm in guiding its design, improvement, and application in practice. The theoretical analysis can be divided into convergence analysis and running-time analysis. Zhou et al. [31], Qiao et al. [32], and Zhang et al. [33] have performed corresponding convergence analyses on BSO. The BSO–CAPSO algorithm, proposed by Alkmini [1], effectively enhances the computational efficiency of BSO through hybridization with chaotic accelerated particle swarm optimization. However, there are few works on the running-time analysis of BSO.

Some theoretical methods have been proposed as general analysis tools to investigate the running time of random heuristic algorithms, including the fitness level method [34], drift analysis method [35], switch analysis method [36], wave model [37], etc. These methods are mainly used to analyze discrete random heuristic algorithms. In contrast, fewer theoretical analysis results have been obtained for continuous random heuristic algorithms [38,39,40]. However, a large number of practical application problems are continuous. Therefore, the running time of continuous random heuristic algorithms has important research significance. To analyze the running time of continuous random heuristic algorithms, Huang et al. [41] proposed the average gain model.

Huang et al. [41,42] and Zhang et al. [43] used an average gain model to evaluate the expected first hit time of the (1+1)-evolutionary algorithm ((1+1) EA), evolutionary strategy (ES), and covariance matrix adaptation evolution strategy (CMA-ES). The concept of the first hit time refers to the minimum number of iterations required before the algorithm finds an optimal solution [35]. The expected first hit time represents the average number of iterations needed to find the optimal solution, which actually reflects the average time complexity of the algorithm [44]. Wang [45] employed the average gain model to analyze the computational efficiency of the proposed swarm intelligence algorithm and provided theoretical evidence for its effectiveness. Therefore, the expected first hit time is a core metric in runtime analysis. Based on the average gain model, the expected first hit time of BSO is deeply analyzed in this paper.

The core of BSO consists of three key components: clustering, interruption, and update. The mutation operator plays an important role in BSO and is included in the interrupt operation and update operation. Specifically, the mutation operator helps the algorithm to jump out of the local optimal solution and further explore a broader search space by introducing random factors in the search process. For example, Zhan et al. [46] proposed an improved BSO (MBSO) in which the mutation operator employs a novel thought difference strategy (IDS). This strategy takes advantage of the thought differences among individuals in the group and increases the diversity of the group by introducing random factors, thus increasing the probability of the algorithm finding the global optimal solution. In addition, El-Abd [47] improved the step equation of the mutation operator and improved the performance of BSO by adjusting the step size and distribution. This improvement helps the algorithm to balance the local search and the global search better, so that the algorithm can find the global optimal solution more effectively when solving complex optimization problems.

The time complexity of the single individual BSO is analyzed in this paper based on the research process from simple to complex. The single individual BSO without the disrupting operation is the same as the (1+1) ES [41]. However, the corresponding results can explain the influence of the mutation operator and disrupting operator on the time complexity of BSO. In this paper, we choose the three most classic and representative distributions, N(0,1), $U\left(-\frac{1}{2},\frac{1}{2}\right)$, and U(−1,1), as the analysis objects for the mutation operator. Therefore, six BSO variants are obtained as the analyzed algorithms based on the combination of three mutation operators and the presence or absence of a disrupting operator.

The remainder of this paper is organized as follows. Section 2 introduces the process of BSO and the mathematical model of running-time analysis for BSO. Section 3 provides the theoretical analysis results for the running-time analysis of three different BSO variants. Section 4 presents the corresponding experimental results to evaluate the theoretical results. Finally, Section 5 concludes the paper.

## 2. Mathematical Model for Running-Time Analysis of BSO

### 2.1. Brain Storm Optimization

BSO was proposed by Shi [48,49] in 2011, and it is simple in concept and easy to implement. BSO can be simplified as follows.

In Steps 4 and 5, the new solution is generated by $\tilde{x}=x+\Delta$, where *x* is the original individual, $\tilde{x}$ is the newly generated individual, and Δ is a vector generated according to the mutation operator. In this paper, we focus on the running time of BSO with three different mutation operators. The same mutation operators are used to generate new individuals in both Steps 4 and 5. The superposition of different mutation operators is not considered in this work.

To accurately observe the effects of the disrupting and updating operations on the running time of BSO, we select a single individual form of BSO as the analyzed object. The single individual BSO framework simplifies the effect of the population size, which helps to evaluate the effect of the disrupting and updating operations on the running time. Furthermore, following the principle from simple to difficult, the single individual BSO is a suitable starting point for the running-time analysis of BSO. Moreover, the randomness of Δ and the design of the operations in Steps 4 and 5 are derived from the mutation operator design of evolutionary programming [50,51]. Therefore, the conclusion of this analysis will have positive implications for the study of similar mutation operators in evolutionary programming algorithms.

### 2.2. Stochastic Process Model of BSO

BSO can be represented as a stochastic process. In this section, we introduce the terminology for the analysis of the running time of BSO.

**Definition** **1**  (Hill-climbing task)**.** Given a search space $S\subseteq R^{n}$ and a fitness function f:S→R, the hill-climbing task is to find a solution ${\vec{x}}^{*}\in S,$ where the fitness of ${\vec{x}}^{*}$ reaches the target value H, where $f({\vec{x}}^{*})\geq H$.

In this paper, we focus on analyzing the BSO running time in the hill-climbing task of a continuous search space.

**Definition** **2**  (State of BSO)**.** *The state of BSO at the t-th (t=0,1,…) iteration is defined as $P_{t}=\left\{{\vec{\xi }}_{1}^{t},{\vec{\xi }}_{2}^{t},\ldots ,{\vec{\xi }}_{\lambda }^{t}\right\}$, where λ is the size of the population, and ${\vec{\xi }}_{1}^{t},{\vec{\xi }}_{2}^{t},\ldots ,{\vec{\xi }}_{\lambda }^{t}\in S$.*

**Definition** **3**  (State space of BSO)**.** *The set of all possible BSO states is called the state space of BSO, denoted as*

(1) $$\Omega =S^{\lambda }=\left\{\left\{{\vec{\xi }}_{1},{\vec{\xi }}_{2},\ldots ,{\vec{\xi }}_{\lambda }\right\}|{\vec{\xi }}_{k}\in S,\;k=1,\ldots ,\lambda \right\}.$$

An optimization problem is a mapping from a decision space to an objective space, and the state space of BSO represents the corresponding decision space.

**Definition** **4**  (Expected first hitting time)**.** *Let ${\left\{X_{t}\right\}}_{t=0}^{\infty }$ be a stochastic process, where, for any t≥0, $X_{t}\geq 0$ holds. Suppose that $X_{t}$ is the Euclidean distance value of the t-th iteration state of BSO to the target solution, and the target threshold ε>0, the first hitting time [35] of the ε-approximation solution, can be defined by*

(2) $$T_{\varepsilon }=\min\left\{t\geq 0:X_{t}\leq \varepsilon \right\}.$$

*Therefore, the expected first hitting time [44] of BSO can be denoted with $E(T_{\varepsilon }|X_{0})$.*

The expected first hit time refers to the average number of iterations required for the BSO algorithm to reach the target fitness value. This metric can more accurately measure the performance of an algorithm because it takes into account the probability distribution of the algorithm over different iterations. Through this metric, we can evaluate the average time complexity of the algorithm in finding the optimal solution, so as to better understand the efficiency of the algorithm.

### 2.3. Running-Time Analysis of BSO Based on Average Gain Model

Inspired by drift analysis [52] and the idea of Riemann integrals [53], Huang et al. [41] proposed the average gain model. Zhang et al. [43] separated this model by introducing the concepts of the supermartingale and stopping time. Based on the former research results [41,43] of the average gain model, Huang et al. [42] proposed an experimental method to estimate the running time of the continuous evolution algorithms.

The expected one-step variation

(3) $$\begin{array}{c} \delta_{t}=E\left(X_{t}-X_{t+1}|H_{t}\right),\;t\geq 0 \end{array}$$

is called the average gain, where ${\left\{X_{t}\right\}}_{t=0}^{\infty }$ is a stochastic process, and $H_{t}=\sigma \left(X_{0},X_{1},\ldots ,X_{t}\right)$. The σ-algebra $H_{t}$ contains all the events generated by $X_{0},X_{1},\ldots ,X_{t}$. All the information observed from the original population to the *t*-th iteration is recorded in $H_{t}$.

Based on Definition 2, $P_{t}=\left\{{\vec{\xi }}_{1}^{t},{\vec{\xi }}_{2}^{t},\ldots ,{\vec{\xi }}_{\lambda }^{t}\right\}$ is the state of BSO at the *t*-th iteration. The process of BSO in solving the hill-climbing task is considered as the gradual process of the stochastic state from the initial population to the population that contains the optimal solution. Let $f\left(P_{t}\right)=\max\left\{f\left(\vec{x}\right):\vec{x}\in P_{t}\right\}$ be the highest fitness value of individuals in $P_{t}$. $X_{t}$ is used to measure the distance of the current population to the population of the target value. $X_{t}=f^{*}-f\left(P_{t}\right)$, where $f^{*}$ is the fitness value of the optimal solution. Obviously, ${\left\{X_{t}\right\}}_{t=0}^{\infty }$ is a non-negative stochastic process.

The state of BSO in the (t+1)-th iteration $P_{t+1}$ only depends on $P_{t}$. In other words, the stochastic process ${\left\{P_{t}\right\}}_{t=0}^{\infty }$ can be modeled by a Markov chain [42]. Similarly, ${\left\{X_{t}\right\}}_{t=0}^{\infty }$ can also be regarded as a Markov chain. In this case, the average gain $\delta_{t}=E\left(X_{t}-X_{t+1}|H_{t}\right)$ can be simplified to $\delta_{t}=E\left(X_{t}-X_{t+1}|X_{t}\right)$. Based on Th. 2 of  [43], the expectation of $T_{\varepsilon }$ of BSO can be estimated as follows.

**Theorem** **1.**  *Suppose that ${\left\{X_{t}\right\}}_{t=0}^{\infty }$ is a stochastic process associated with BSO, where $X_{t}\geq 0$ for all t≥0. Let $h:\left[0,A\right]\to R^{+}$ be a monotonically increasing and integrable function. If $E\left(X_{t}-X_{t+1}|X_{t}\right)\geq h\left(X_{t}\right)$ and $X_{t}>\varepsilon >0$, it holds for $T_{\varepsilon }$ that*

(4) $$E\left(T_{\varepsilon }|X_{0}\right)\leq 1+\int_{\varepsilon }^{X_{0}}\frac{1}{h(x)}x.$$

Theorem 1 shows the upper bounds on the expected first hitting time of BSO based on the average gain model. The average gain $\delta_{t}$ plays a key role in analyzing the first hitting time $T_{\varepsilon }$ of the ε-approximation solution for BSO. The higher average gain indicates a more efficient iteration of the optimization process.

## 3. Running-Time Analysis of BSO Instances for Equal Coefficient Linear Functions

In this section, we present the theoretical analysis results based on the average gain model to analyze the expected first hitting time of BSO for equal coefficient linear functions. The running time of BSO with three different mutation operators is analyzed from the perspective of whether the disrupting operation exists. In this paper, we refer to the BSO without a disrupting operation as BSO-I, and the BSO with a disrupting operation as BSO-II.

On this basis, the equal coefficient linear functions are selected as the research object [54,55,56]. These functions are a form of basic continuous optimization problem whose function expression is as follows:

(5) $$\begin{array}{c} f\left(x_{1},x_{2}\ldots ,x_{n}\right)=k\left(x_{1}+x_{2}+\ldots +x_{n}\right)=k\sum_{i=1}^{n}x_{i}, \end{array}$$

where $(x_{1},x_{2},\ldots ,x_{n})\in S$. It is assumed that the function starts from the origin and sets the target fitness value to na, where a>0. The objective of optimizing the equal coefficient linear function is to find a solution ${\vec{x}}^{*}\in S$, such that $f({\vec{x}}^{*})\geq na$.

The mutation operator that obeys the Gaussian distribution and the uniform distribution is selected for the evaluation of BSO. The Gaussian distribution and uniform distribution are common tools for the design of mutation operators [50,51,57], so it is representative to select these two distributions as research cases.

### 3.1. Case Study of BSO without Disrupting Operator

Since the single individual BSO is analyzed in this paper, λ is equal to 1 in the state $P_{t}=\left\{\xi_{1}^{t},\xi_{2}^{t},\ldots ,\xi_{\lambda }^{t}\right\}$ of BSO. The BSO of a single individual has only one individual, so $\xi_{1}^{t}$ represents both the optimized individual and the random state of the algorithm. The procedure of single individual BSO can be described as follows when the disrupting operation does not exist (i.e., Step 4 in Algorithm 1 is ignored).
**Algorithm 1** Brain Storm Optimization (BSO)1:**Initialization**: Randomly generate λ individuals (potential solutions) to form the initial population $P=\{\xi_{1},\xi_{2},\ldots ,\xi_{\lambda }\}$ and evaluate the λ individuals;2:**while** fail to achieve the predetermined maximum number of iterations **do**3:   **Clustering**: Use clustering algorithms to divide λ individuals into *m* clusters;4:   **Disrupting**: The mutation occurs with a certain probability, and a randomly selected cluster’s central individual is replaced by a randomly generated new individual;5:   **Updating**: Randomly choose one or two clusters to create a new individual;   Compare the newly generated individual and the original individual with the same individual index. The better one will be saved as the new individual;   Update the whole population; the offspring population is recorded as $P^{\prime }=\left\{\xi_{1}^{\prime },\xi_{2}^{\prime },\ldots ,\xi_{\lambda }^{\prime }\right\}$. Evaluate the individuals in $P^{\prime }$;6:**end while**7:Output the most optimal solution discovered.


${\vec{x}}^{t}=\left(x_{1}^{t},x_{2}^{t},\ldots ,x_{n}^{t}\right)\in S,\;t=0,1,\ldots$ is the *t*-th generation of the algorithm.

(6) $$\begin{array}{c} X_{t}=an-f\left({\vec{x}}^{t}\right)=an-k\left(x_{1}^{t}+x_{2}^{t},\ldots +x_{n}^{t}\right), \end{array}$$

is defined as the Euclidean distance of the *t*-th iteration to the optimal solution. The gain at *t*-th is given by

(7) $$\begin{array}{cc} \eta_{t} & =X_{t}-X_{t+1}\\ & =k\left(x_{1}^{t+1}+x_{2}^{t+1}+\ldots +x_{n}^{t+1}\right)-k\left(x_{1}^{t}+x_{2}^{t}+\ldots +x_{n}^{t}\right). \end{array}$$

#### 3.1.1. When $z_{i}∼N\left(0,1\right)$

If the mutation operator obeys the standard normal distribution N(0,1), the distribution function of $\eta_{t}$ is as presented by Lemma 1.

**Lemma** **1.**  *For BSO-I, if its mutation operator $\vec{z}$ obeys N(0,1), the distribution function $F\left(u\right)=P\left(\eta_{t}\leq u\right)$ of the gain $\eta_{t}$ is*

(8) $$F\left(u\right)=\left\{\begin{array}{cc} 0, & u<0\\ \frac{1}{2}, & u=0\\ \frac{1}{\sqrt{2\pi n}k}\int_{-\infty }^{u}e^{-\frac{t^{2}}{2nk^{2}}}t, & u>0 \end{array}\right..$$

**Proof.**  According to Step 4 and Step 5 of Algorithm 2, the (t+1)-th individual is

(9) $$\begin{array}{c} {\vec{x}}^{t+1}=\left\{\begin{array}{cc} {\vec{x}}^{t}, & f\left({\vec{y}}^{t}\right)\leq f\left({\vec{x}}^{t}\right)\\ {\vec{y}}^{t}, & f\left({\vec{y}}^{t}\right)>f\left({\vec{x}}^{t}\right) \end{array}\right.. \end{array}$$

**Algorithm 2** BSO-I1:Initialization: Randomly generate an individual $\vec{x}=\left(x_{1},x_{2},\ldots ,x_{n}\right)\in R^{n}$ based on uniform distribution;2:**while** stopping criterion is not satisfied **do**3:   $\vec{y}=\vec{x}+\vec{z}$, where $\vec{z}$ is the mutation operator;4:   **if** $\vec{y}$ adapts better than $\vec{x}$ **then**5:      $\vec{x}$ is substituted for $\vec{y}$6:   **end if**7:**end while****Output:** 
$\vec{x}$

According to the definition of $\eta_{t}$, where t=0,1,…,

(1) If $f\left({\vec{y}}^{t}\right)\leq f\left({\vec{x}}^{t}\right)$,

(10) $$\begin{array}{cc} \eta_{t} & =k\left(x_{1}^{t+1}+x_{2}^{t+1}+\ldots +x_{n}^{t+1}\right)-k\left(x_{1}^{t}+x_{2}^{t}+\ldots +x_{n}^{t}\right)\\ & =k\left(x_{1}^{t}+x_{2}^{t}+\ldots +x_{n}^{t}\right)-k\left(x_{1}^{t}+x_{2}^{t}+\ldots +x_{n}^{t}\right)=0 \end{array}$$

(2) If $f\left({\vec{y}}^{t}\right)>f\left({\vec{x}}^{t}\right)$,

(11) $$\begin{array}{cc} \eta_{t} & =k\left(x_{1}^{t+1}+x_{2}^{t+1}+\ldots +x_{n}^{t+1}\right)-k\left(x_{1}^{t}+x_{2}^{t}+\ldots +x_{n}^{t}\right)\\ & =k\left(y_{1}^{t}+y_{2}^{t}+\ldots +y_{n}^{t}\right)-k\left(x_{1}^{t}+x_{2}^{t}+\ldots +x_{n}^{t}\right)\\ & =k[\left(y_{1}^{t}-x_{1}^{t}\right)+\left(y_{2}^{t}-x_{2}^{t}\right)+\ldots +\left(y_{n}^{t}-x_{n}^{t}\right)]\\ & =k\left(z_{1}^{t}+z_{2}^{t}+\ldots +z_{n}^{t}\right)=f\left({\vec{z}}^{t}\right) \end{array}$$

Since $z_{i}∼N\left(0,1\right)$, $z_{1},\ldots ,z_{n}$ are independent of each other. All of the $z_{i}$ satisfy the additivity of the normal distribution, so $f({\vec{z}}^{t})$ obeys the distribution of $N(0,nk^{2})$.

Hence, the distribution function of $\eta_{t}$ is shown as $F\left(u\right)=P\left(\eta_{t}\leq u\right)$.

(1) If u<0, according to the definition of $\eta_{t}$ where $\eta_{t}\geq 0$, it has F(u)=0.
(2) If u=0, the probability density function of N(0,n) is symmetric in the *y* axis, so $F\left(u\right)=P\left(\eta_{t}\leq u\right)=P\left(\eta_{t}=0\right)=\frac{1}{2}$.
(3) If u>0, $F\left(u\right)=P\left(\eta_{t}\leq u\right)=\frac{1}{\sqrt{2\pi n}k}\int_{-\infty }^{u}e^{-\frac{t^{2}}{2nk^{2}}}t$.

Lemma 1 holds.    □

Theorem 2 is presented based on the above proof.

**Theorem** **2.**  *If the mutation operator $\vec{z}$ of BSO-I obeys N(0,1), the upper bound on the expected first hitting time to reach the target fitness value na is derived as follows.*

(12) $$\begin{array}{c} E\left(T_{\varepsilon }|X_{0}\right)\leq 1+\sqrt{2\pi n}\frac{a}{k}-\sqrt{\frac{2\pi }{n}}\frac{\varepsilon }{k}. \end{array}$$

**Proof.**  (13) $$\begin{array}{c} E\left(X_{t}-X_{t+1}|X_{t}\right)=E\left(\eta_{t}|X_{t}\right)=\int_{-\infty }^{+\infty }uF\left(u\right)\\ =\int_{0}^{+\infty }u\left(\frac{1}{\sqrt{2\pi n}k}\int_{-\infty }^{u}e^{-\frac{t^{2}}{2nk^{2}}}t\right)=\frac{k\sqrt{n}}{\sqrt{2\pi }} \end{array}$$

It is assumed that the algorithm starts from the origin at initialization, where ${\vec{x}}^{0}=\left(0,0,\ldots ,0\right)$, i.e.,

(14) $$\begin{array}{c} X_{0}=na-f\left({\vec{x}}^{0}\right)=na-k\left(0+0\ldots +0\right)=na \end{array}$$

According to Theorem 1, the upper bound on the expected first hitting time is derived as

(15) $$\begin{array}{c} E\left(T_{\varepsilon }|X_{0}\right)\leq 1+\int_{\varepsilon }^{na}\frac{\sqrt{2\pi }}{k\sqrt{n}}x=1+\sqrt{2\pi n}\frac{a}{k}-\sqrt{\frac{2\pi }{n}}\frac{\varepsilon }{k}. \end{array}$$

Theorem 2 holds.    □

Theorem 2 indicates that for BSO-I, if its mutation operator $\vec{z}$ obeys N(0,1), the computational time complexity of BSO-I for the equal coefficient linear function is $E\left(T_{\varepsilon }|X_{0}\right)=O\left(\sqrt{n}\right)$.

#### 3.1.2. When $z_{i}∼U\left(-\frac{1}{2},\frac{1}{2}\right)$

The uniform distribution function does not satisfy additivity like the normal distribution function. The Lindeberg–Levy center limit theorem [58] can provide an idea to find the distribution of $\eta_{t}$. The Lindeberg–Levy center limit theorem is introduced below.

Suppose that $X_{n}$ is a sequence of independent and identically distributed random variables with $E(X_{i})=\mu$ and $\left(X_{i}\right)=\sigma^{2}>0$; let

(16) $$\begin{array}{c} Y_{n}^{*}=\frac{X_{1}+X_{2}+\ldots +X_{n}-n\mu }{\sigma \sqrt{n}}, \end{array}$$

then

(17) $$\begin{array}{c} \lim_{n\to \infty }P\left(Y_{n}^{*}\leq y\right)=\Phi \left(y\right)=\frac{1}{\sqrt{2\pi }}\int_{-\infty }^{y}e^{-\frac{t^{2}}{2}}t \end{array}$$

is satisfied for any real number *y*.

The Lindeberg–Levy center limit theorem [58] shows that if *n* is sufficiently large, $Y_{n}^{*}∼N\left(0,1\right)$, it has $\sum_{i=1}^{n}X_{i}∼N\left(n\mu ,n\sigma^{2}\right)$. Generally, the case of higher dimensions requires more attention in the study of the computational time complexity of algorithms. If the mutation operator obeys $U\left(-\frac{1}{2},\frac{1}{2}\right)$, the distribution function of $\eta_{t}$ can be represented by Lemma 2.

**Lemma** **2.**  *For BSO-I, if its mutation operator $\vec{z}$ obeys $U\left(-\frac{1}{2},\frac{1}{2}\right)$, the distribution function $F\left(u\right)=P\left(\eta_{t}\leq u\right)$ of the gain $\eta_{t}$ is*

(18) $$\begin{array}{c} F\left(u\right)=\left\{\begin{array}{cc} 0, & u<0\\ \frac{1}{2}, & u=0\\ \frac{\sqrt{6}}{\sqrt{\pi n}k}\int_{-\infty }^{u}e^{-\frac{6t^{2}}{nk^{2}}}t, & u>0 \end{array}\right.. \end{array}$$

**Proof.**  According to the definition of $\eta_{t}$, where t=0,1,….

(1) If $f\left({\vec{y}}^{t}\right)\leq f\left({\vec{x}}^{t}\right),\eta_{t}=0$.
(2) If $f\left({\vec{y}}^{t}\right)>f\left({\vec{x}}^{t}\right),\eta_{t}=k\left(z_{1}^{t}+z_{2}^{t}+\ldots +z_{n}^{t}\right)$, where $z_{i}∼U\left(-\frac{1}{2},\frac{1}{2}\right)$, and $z_{1},\ldots ,z_{n}$ are independent of each other. According to the Lindeberg–Levy center limit theorem, $\eta_{t}$ obeys $N\left(0,\frac{1}{12}nk^{2}\right)$.

Hence, the distribution function of $\eta_{t}$ is $F\left(u\right)=P\left(\eta_{t}\leq u\right)$.

(1) If u<0, F(u)=0.
(2) If u=0, $F\left(u\right)=P\left(\eta_{t}=0\right)=\frac{1}{2}$.
(3) If u>0, $F\left(u\right)=\frac{\sqrt{6}}{\sqrt{\pi n}k}\int_{-\infty }^{u}e^{-\frac{6t^{2}}{nk^{2}}}t$.

Lemma 2 holds.    □

Theorem 3 is presented based on the above proof.

**Theorem** **3.**  *For BSO-I, if its mutation operator $\vec{z}$ obeys $U\left(-\frac{1}{2},\frac{1}{2}\right)$, the upper bound on the expected first hitting time to reach the target fitness value na is derived as follows.*

(19) $$\begin{array}{c} E\left(T_{\varepsilon }|X_{0}\right)\leq 1+2\sqrt{6\pi n}\frac{a}{k}-\frac{2\sqrt{6\pi }}{\sqrt{n}}\frac{\varepsilon }{k}. \end{array}$$

The proof of this theorem is based on the same principle as Theorem 2. The detailed derivation is given as follows.

**Proof.**  According to Lemma 2, we have

(20) $$\begin{array}{cc} \; & E\left(X_{t}-X_{t+1}|X_{t}\right)=E\left(\eta_{t}|X_{t}\right)=\int_{-\infty }^{+\infty }uF\left(u\right)\\ \; & =\int_{0}^{+\infty }u\left(\frac{\sqrt{6}}{\sqrt{\pi n}k}\int_{-\infty }^{u}e^{-\frac{6t^{2}}{nk^{2}}}t\right)=\frac{k\sqrt{n}}{2\sqrt{6\pi }}, \end{array}$$

and the algorithm starts from the origin at initialization, ${\vec{x}}^{0}=\left(0,0,\ldots ,0\right)$, i.e., $X_{t}=na$. According to Theorem 1, the upper bound on the expected first hitting time is derived as

(21) $$\begin{array}{c} E\left(T_{\varepsilon }|X_{0}\right)\leq 1+\int_{\varepsilon }^{na}\frac{2\sqrt{6\pi }}{k\sqrt{n}}x=1+2\sqrt{6\pi n}\frac{a}{k}-\frac{2\sqrt{6\pi }}{\sqrt{n}}\frac{\varepsilon }{k}.\;\;\;□ \end{array}$$

Theorem 3 indicates that if the mutation operator $\vec{z}$ of BSO-I obeys $U\left(-\frac{1}{2},\frac{1}{2}\right)$, its computational time complexity is $E\left(T_{\varepsilon }|X_{0}\right)=O\left(\sqrt{n}\right)$ for the equal coefficient linear function.

#### 3.1.3. When $z_{i}∼U\left(-1,1\right)$

If the mutation operator obeys U(−1,1), the distribution function of $\eta_{t}$ can be represented by Lemma 3.

**Lemma** **3.**  *For BSO-I, if its mutation operator $\vec{z}$ obeys U(−1,1), the distribution function $F\left(u\right)=P\left(\eta_{t}\leq u\right)$ of the gain $\eta_{t}$ is*

(22) $$\begin{array}{c} F\left(u\right)=\left\{\begin{array}{cc} 0, & u<0\\ \frac{1}{2}, & u=0\\ \frac{\sqrt{3}}{\sqrt{2\pi nk}}\int_{-\infty }^{u}e^{-\frac{3t^{2}}{2nk^{2}}}t, & u>0 \end{array}\right.. \end{array}$$

The proof of this lemma is based on the same principle as Lemma 2.

**Proof.**  According to the definition of $\eta_{t},t=0,1,\ldots$,

(1) If $f\left({\vec{y}}^{t}\right)\leq f\left({\vec{x}}^{t}\right),\eta_{t}=0$.
(2) If $f\left({\vec{y}}^{t}\right)>f\left({\vec{x}}^{t}\right),\eta_{t}=f\left({\vec{z}}^{t}\right)$.

Since $z_{i}∼U\left(-1,1\right)$, $z_{1},\ldots ,z_{n}$ are independent of each other, according to the Lindeberg–Levy center limit theorem, $f({\vec{z}}^{t})$ obeys $N(0,\frac{1}{3}nk^{2})$.

Hence, the $\eta_{t}$ distribution function $F\left(u\right)=P\left(\eta_{t}\leq u\right)$ is

(1) If u<0, F(u)=0.
(2) If u=0, $F\left(u\right)=P\left(\eta_{t}=0\right)=\frac{1}{2}$.
(3) If u>0, $F\left(u\right)=\frac{\sqrt{3}}{\sqrt{2\pi nk}}\int_{-\infty }^{u}e^{-\frac{3t^{2}}{2nk^{2}}}t$.

   □

According to Lemma 3 and Theorem 1, Theorem 4 can be inferred.

**Theorem** **4.**  *For BSO-I, if its mutation operator $\vec{z}$ obeys U(−1,1), the upper bound on the expected first hitting time to reach the target fitness value na is derived as*

(23) $$\begin{array}{c} E\left(T_{\varepsilon }|X_{0}\right)\leq 1+\sqrt{6\pi n}\frac{a}{k}-\sqrt{\frac{6\pi }{n}}\frac{\varepsilon }{k}. \end{array}$$

The proof of this theorem is based on the same principle as Theorem 2. The proof is given as follows.

**Proof.**  According to Lemma 3, we have

(24) $$\begin{array}{c} E\left(X_{t}-X_{t+1}|X_{t}\right)=E\left(\eta_{t}|X_{t}\right)=\int_{-\infty }^{+\infty }uF\left(u\right)\\ =\int_{0}^{+\infty }u\left(\frac{\sqrt{3}}{\sqrt{2\pi nk}}\int_{-\infty }^{u}e^{-\frac{3t^{2}}{2nk^{2}}}t\right)=\frac{k\sqrt{n}}{\sqrt{6\pi }}, \end{array}$$

and the algorithm starts from the origin at initialization, ${\vec{x}}^{0}=\left(0,0,\ldots ,0\right)$, i.e., $X_{t}=na$. According to Theorem 1, the upper bound on the expected first hitting time is derived as

(25) $$\begin{array}{c} E\left(T_{\varepsilon }|X_{0}\right)\leq 1+\int_{\varepsilon }^{na}\frac{\sqrt{6\pi }}{k\sqrt{n}}x=1+\sqrt{6\pi n}\frac{a}{k}-\sqrt{\frac{6\pi }{n}}\frac{\varepsilon }{k}.\;\;\;□ \end{array}$$

Theorem 4 indicates that if the mutation operator $\vec{z}$ of BSO-I obeys U(−1,1), its computational time complexity for the equal coefficient linear function is $E\left(T_{\varepsilon }|X_{0}\right)=O\left(\sqrt{n}\right)$.

The time complexity of BSO-I with three different mutation operators is $O(\sqrt{n})$. In the next section, we will discuss the running time of BSO considering the case with a disrupting operation.

### 3.2. Case Study of BSO with Disrupting Operator

Based on the average gain model, this section analyzes the upper bounds of the expected first hit time in three BSO cases. When interference operations are added to the single individual BSO, the algorithm process can be simplified as follows.

In Algorithm 3, the disrupting operations, which are executed with a small probability, are shown in Steps 3 to 6. Let $A=\{Pb^{\prime }|Pb^{\prime }<Pb\}$ indicate that replacement occurs, while $\bar{A}=\left\{Pb^{\prime }|Pb^{\prime }\geq Pb\right\}$ indicates that no replacement occurs.

**Algorithm 3** BSO-II
1:Initialization: Randomly generate an individual $\vec{x}=\left(x_{1},x_{2},\ldots ,x_{n}\right)\in R^{n}$ based on uniform distribution;2:**while** stopping criterion is not satisfied **do**3:   Randomly generate a value $Pb^{\prime }$ from 0 to 1 based on uniform distribution;4:   **if** $Pb^{\prime }$ is smaller than a pre-determined probability Pb **then**5:       replace $\vec{x}$ with a randomly generated individual $\vec{b}=\left(b_{1},b_{2},\ldots ,b_{n}\right)$ based on uniform distribution;6:   **end if**7:   **if** $Pb^{\prime }<Pb$ **then**8:       $\vec{y}=\vec{b}+\vec{z}$, where $\vec{z}$ is a mutation operator;9:   **else**10:     $\vec{y}=\vec{x}+\vec{z}$;11:  **end if**12:  **if** $\vec{y}$ has better fitness than $\vec{x}$ **then**13:      replace $\vec{x}$ with $\vec{y}$14:  **end if**15:
**end while**
**Output:** 


$\vec{x}$




To highlight the effect of each mutation operator on the algorithm, we choose the same mutation operators of BSO-I in Steps 5 and 8 to generate new individuals. As a result, $b_{i}=x_{i}+\Delta x_{i}$, where mutation operator parameter $\Delta x_{i}$ and $z_{i}$ follow the same distribution.

Here, ${\vec{x}}^{t}=\left(x_{1}^{t},x_{2}^{t},\ldots ,x_{n}^{t}\right)\in S$ is still the *t*-th individual of the algorithm. We have $X_{t}=an-f\left({\vec{x}}_{t}\right)=an-k\left(x_{1}^{t}+x_{2}^{t},\ldots +x_{n}^{t}\right)$, and the corresponding gain at *t* is given by $\eta_{t}=X_{t}-X_{t+1}=k\left(x_{1}^{t+1}+x_{2}^{t+1}+\ldots +x_{n}^{t+1}\right)-k\left(x_{1}^{t}+x_{2}^{t}+\ldots +x_{n}^{t}\right)$.

#### 3.2.1. When $z_{i}∼N\left(0,1\right)$

(1) If $Pb^{\prime }\geq Pb$, it is the same as the result of the case with no disrupting operation in Section 3.1, and the average gain is

(26) $$\begin{array}{c} E\left(X_{t}-X_{t+1}|X_{t},\bar{A}\right)=E\left(\eta_{t}|X_{t},\bar{A}\right)=\frac{k\sqrt{n}}{\sqrt{2\pi }}. \end{array}$$

(2) If $Pb^{\prime }<Pb$ and the mutation operator obeys N(0,1), the distribution function of $\eta_{t}$ is represented by Lemma 4.

**Lemma** **4.**  *For BSO-II, if its mutation operator $\vec{z}$ obeys N(0,1) and $Pb^{\prime }<Pb$, the distribution function of the gain $\eta_{t}$ is $F\left(u\right)=P\left(\eta_{t}\leq u\right)$.*

(27) $$\begin{array}{c} F\left(u\right)=\left\{\begin{array}{cc} 0, & u<0\\ \frac{1}{2}, & u=0\\ \frac{1}{2k\sqrt{\pi n}}\int_{-\infty }^{u}e^{-\frac{t^{2}}{4nk^{2}}}t, & u>0 \end{array}\right.. \end{array}$$

**Proof.**  According to Step 12 and Step 13 of Algorithm 3, the (t+1)-th individual is ${\vec{x}}^{t+1}=\left\{\begin{array}{cc} {\vec{x}}^{t}, & f\left({\vec{y}}^{t}\right)\leq f\left({\vec{x}}^{t}\right)\\ {\vec{y}}^{t}, & f\left({\vec{y}}^{t}\right)>f\left({\vec{x}}^{t}\right) \end{array}\right.$.

According to the definition of $\eta_{t},t=0,1,\ldots$,

(1) If $f\left({\vec{y}}^{t}\right)\leq f\left({\vec{x}}^{t}\right),\;\eta_{t}=0$.
(2) If $f\left({\vec{y}}^{t}\right)>f\left({\vec{x}}^{t}\right)$,

(28) $$\begin{array}{cc} \eta_{t} & =k\left(x_{1}^{t+1}+x_{2}^{t+1}+\ldots +x_{n}^{t+1}\right)-k\left(x_{1}^{t}+x_{2}^{t}+\ldots +x_{n}^{t}\right)\\ \; & =k\left[\left(b_{1}^{t}+b_{2}^{t}+\ldots +b_{n}^{t}\right)+\left(z_{1}^{t}+z_{2}^{t}+\ldots +z_{n}^{t}\right)\right]-k\left(x_{1}^{t}+x_{2}^{t}+\ldots +x_{n}^{t}\right)\\ \; & =k[\left(x_{1}^{t}+x_{2}^{t}+\ldots +x_{n}^{t}\right)+\left(\Delta x_{1}^{t}+\Delta x_{2}^{t}+\ldots +\Delta x_{n}^{t}\right)]\\ \; & \;+k\left(z_{1}^{t}+z_{2}^{t}+\ldots +z_{n}^{t}\right)-k\left(x_{1}^{t}+x_{2}^{t}+\ldots +x_{n}^{t}\right)\\ \; & =k[\left(\Delta x_{1}^{t}+\Delta x_{2}^{t}+\ldots +\Delta x_{n}^{t}\right)+\left(z_{1}^{t}+z_{2}^{t}+\ldots +z_{n}^{t}\right)] \end{array}$$

Since $z_{i}∼N\left(0,1\right)$, $\Delta x_{i}∼N\left(0,1\right)$, and $z_{1},\ldots ,z_{n}$ are independent of each other, $\Delta x_{1},\Delta x_{2},\ldots ,\Delta x_{n}$ are also independent of each other. All of $z_{i}$ and $\Delta x_{i}$ satisfy the additivity of the normal distribution. As a result, $\eta_{t}$ obeys $N(0,2nk^{2})$.

Hence, the distribution function of $\eta_{t}$ is $F\left(u\right)=P\left(\eta_{t}\leq u\right)$.

(1) If u<0, F(u)=0.
(2) If u=0, $F\left(u\right)=P\left(\eta_{t}\leq u\right)=P\left(\eta_{t}=0\right)=\frac{1}{2}$.
(3) If u>0, $F\left(u\right)=P\left(\eta_{t}\leq u\right)=\frac{1}{2k\sqrt{\pi n}}\int_{-\infty }^{u}e^{-\frac{t^{2}}{4nk^{2}}}t$.

Lemma 4 holds. □

Based on the above proofs, we can conclude that

(29) $$\begin{array}{c} E\left(X_{t}-X_{t+1}|X_{t},A\right)=E\left(\eta_{t}|X_{t},A\right)=\int_{0}^{+\infty }u\left(\frac{1}{2k\sqrt{\pi n}}\int_{-\infty }^{u}e^{-\frac{t^{2}}{4nk^{2}}}t\right)=\frac{k\sqrt{n}}{\sqrt{\pi }} \end{array}$$

Assume that the probability Pb is equal to 0.2 [59]; Theorem 5 can be obtained according to Theorem 1.

**Theorem** **5.**  *For BSO-II, if its mutation operator $\vec{z}$ obeys N(0,1), the upper bound on the expected first hitting time to reach the target fitness value na is derived as follows.*

(30) $$\begin{array}{c} E\left(T_{\varepsilon }|X_{0}\right)\leq 1+\frac{5\sqrt{2\pi n}}{4+\sqrt{2}}\frac{a}{k}-\frac{5\sqrt{2\pi }}{4\sqrt{n}+\sqrt{2n}}\frac{\varepsilon }{k}. \end{array}$$

**Proof.** 
(31) $$\begin{array}{cc} E(X_{t}-X_{t+1}|X_{t}) & =P\left(\bar{A}\right)E\left(X_{t}-X_{t+1}|X_{t},\bar{A}\right)+P\left(A\right)E\left(X_{t}-X_{t+1}|X_{t},A\right)\\ \; & \left(\mathrm{full}\;\mathrm{expectation}\;\mathrm{formula}\right)\\ \; & =\left(1-0.2\right)\times E\left(X_{t}-X_{t+1}|X_{t},\bar{A}\right)+0.2\times E\left(X_{t}-X_{t+1}|X_{t},A\right)\\ \; & =0.8\times \frac{k\sqrt{n}}{\sqrt{2\pi }}+0.2\times \frac{k\sqrt{n}}{\sqrt{\pi }}=\frac{4k\sqrt{n}+k\sqrt{2n}}{5\sqrt{2\pi }} \end{array}$$

The algorithm starts from the origin at initialization, ${\vec{x}}^{0}=\left(0,0,\ldots ,0\right)$, i.e., $X_{t}=na$. According to Theorem 1, the upper bound on the expected first hitting time is derived as

(32) $$\begin{array}{cc} E(T_{\varepsilon }|X_{0}) & \leq 1+\int_{\varepsilon }^{na}\frac{5\sqrt{2\pi }}{4k\sqrt{n}+k\sqrt{2n}}x\\ \; & =1+\frac{5\sqrt{2\pi n}}{4+\sqrt{2}}\frac{a}{k}-\frac{5\sqrt{2\pi }}{4\sqrt{n}+\sqrt{2n}}\frac{\varepsilon }{k} \end{array}$$

Theorem 5 holds. □

According to the proof of Theorem 5, if the mutation operator $\vec{z}$ of BSO-II obeys N(0,1), its computational time complexity for the equal coefficient linear function is $E\left(T_{\varepsilon }|X_{0}\right)=O\left(\sqrt{n}\right)$.

#### 3.2.2. When $z_{i}∼U\left(-\frac{1}{2},\frac{1}{2}\right)$

(1) If $Pb^{\prime }\geq Pb$, the result is the same as the case in Section 3.1 with no disrupting operation. The average gain is

(33) $$\begin{array}{c} E\left(X_{t}-X_{t+1}|X_{t},\bar{A}\right)=E\left(\eta_{t}|X_{t},\bar{A}\right)=\frac{k\sqrt{n}}{2\sqrt{6\pi }}. \end{array}$$

(2) If $Pb^{\prime }<Pb$ and the mutation operator obeys $U\left(-\frac{1}{2},\frac{1}{2}\right)$, the distribution function of $\eta_{t}$ is represented by Lemma 5.

**Lemma** **5.**  *If the mutation operator of BSO-II $\vec{z}$ obeys $U\left(-\frac{1}{2},\frac{1}{2}\right)$ and $Pb^{\prime }<Pb$, the distribution function of the gain $\eta_{t}$ is $F\left(u\right)=P\left(\eta_{t}\leq u\right)$.*

(34) $$\begin{array}{c} F\left(u\right)=\left\{\begin{array}{cc} 0, & u<0\\ \frac{1}{2}, & u=0\\ \frac{\sqrt{3}}{\sqrt{\pi nk}}\int_{-\infty }^{u}e^{-\frac{3t^{2}}{nk^{2}}}t, & u>0 \end{array}\right.. \end{array}$$

The proof of this lemma is based on the same principle as Lemma 4. The detailed derivation is given as follows.

**Proof.**  According to the definition of $\eta_{t},t=0,1,\ldots$,

(1) If $f\left({\vec{y}}^{t}\right)\leq f\left({\vec{x}}^{t}\right),\eta_{t}=0$.
(2) If $f\left({\vec{y}}^{t}\right)>f\left({\vec{x}}^{t}\right),\eta_{t}=k\left[\left(\Delta x_{1}^{t}+\Delta x_{2}^{t}+\ldots +\Delta x_{n}^{t}\right)+\left(z_{1}^{t}+z_{2}^{t}+\ldots +z_{n}^{t}\right)\right]$

Since $z_{i}∼U\left(-\frac{1}{2},\frac{1}{2}\right)$, $\Delta x_{i}∼U\left(-\frac{1}{2},\frac{1}{2}\right)$, and $z_{1},\ldots ,z_{n}$ are independent of each other, $\Delta x_{1},\Delta x_{2},\ldots ,\Delta x_{n}$ are also independent of each other. $f({\vec{z}}^{t})$ obeys $N\left(0,\frac{1}{6}nk^{2}\right)$ according to the Lindeberg–Levy center limit theorem.

Hence, the $\eta_{t}$ distribution function $F\left(u\right)=P\left(\eta_{t}\leq u\right)$ is

(1) If u<0, F(u)=0.
(2) If u=0, $F\left(u\right)=P\left(\eta_{t}\leq u\right)=P\left(\eta_{t}=0\right)=\frac{1}{2}$.
(3) If u>0, $F\left(u\right)=P\left(\eta_{t}\leq u\right)=\frac{\sqrt{3}}{\sqrt{\pi n}k}\int_{-\infty }^{u}e^{-\frac{3t^{2}}{nk^{2}}}t$.

□

Theorem 6 can be concluded based on Lemma 5 and Theorem 1.

**Theorem** **6.**  *If the mutation operator of BSO-II $\vec{z}$ obeys $U\left(-\frac{1}{2},\frac{1}{2}\right)$, the upper bound on the expected first hitting time to reach the target fitness value na is derived as follows.*

(35) $$\begin{array}{c} E\left(T_{\varepsilon }|X_{0}\right)\leq 1+\frac{10\sqrt{6\pi n}}{4+\sqrt{2}}\frac{a}{k}-\frac{10\sqrt{6\pi }}{4\sqrt{n}+\sqrt{2n}}\frac{\varepsilon }{k}. \end{array}$$

The proof of this theorem is based on the same principle as Theorem 5. The detailed derivation is presented as follows.

**Proof.**  According to Lemma 5, we have

(36) $$\begin{array}{cc} \; & E\left(X_{t}-X_{t+1}|X_{t},A\right)=E\left(\eta_{t}|X_{t},A\right)=\int_{0}^{+\infty }u\left(\frac{\sqrt{3}}{\sqrt{\pi n}k}\int_{-\infty }^{u}e^{-\frac{3t^{2}}{nk^{2}}}t\right)=\frac{k\sqrt{n}}{2\sqrt{3\pi }}. \end{array}$$

Suppose that the probability Pb=0.2; according to Theorem 1, the following conclusions can be drawn:

(37) $$\begin{array}{cc} \; & E(X_{t}-X_{t+1}|X_{t})\\ \; & =P\left(\bar{A}\right)E\left(X_{t}-X_{t+1}|X_{t},\bar{A}\right)+P\left(A\right)E\left(X_{t}-X_{t+1}|X_{t},A\right)\\ \; & =\left(1-0.2\right)\times E\left(X_{t}-X_{t+1}|X_{t},\bar{A}\right)+0.2\times E\left(X_{t}-X_{t+1}|X_{t},A\right)\\ \; & =0.8\times \frac{k\sqrt{n}}{2\sqrt{6\pi }}+0.2\times \frac{k\sqrt{n}}{2\sqrt{3\pi }}=\frac{4k\sqrt{n}+k\sqrt{2n}}{10\sqrt{6\pi }} \end{array}$$

The algorithm starts from the origin at initialization, ${\vec{x}}^{0}=\left(0,0,\ldots ,0\right)$, i.e., $X_{t}=na$. According to Theorem 1, the upper bound on the expected first hitting time is derived as

(38) $$\begin{array}{c} E\left(T_{\varepsilon }|X_{0}\right)\leq 1+\int_{\varepsilon }^{na}\frac{10\sqrt{6\pi }}{4\sqrt{n}+\sqrt{2n}}x=1+\frac{10\sqrt{6\pi n}}{4+\sqrt{2}}\frac{a}{k}-\frac{10\sqrt{6\pi }}{4\sqrt{n}+\sqrt{2n}}\frac{\varepsilon }{k}.\;\;\;□ \end{array}$$

Theorem 6 indicates that for BSO-II, if its mutation operator $\vec{z}$ obeys $U\left(-\frac{1}{2},\frac{1}{2}\right)$, the computational time complexity of BSO-II for the equal coefficient linear function is $E\left(T_{\varepsilon }|X_{0}\right)=O\left(\sqrt{n}\right)$.

#### 3.2.3. When $z_{i}∼U\left(-1,1\right)$

(1) If $Pb^{\prime }\geq Pb$, the result is the same as the case in Section 3.1 with no disrupting operation. The average gain is

(39) $$\begin{array}{c} E\left(X_{t}-X_{t+1}|X_{t},\bar{A}\right)=E\left(\eta_{t}|X_{t},\bar{A}\right)=\frac{k\sqrt{n}}{\sqrt{6\pi }}. \end{array}$$

(2) If $Pb^{\prime }<Pb$, and the mutation operator obeys U(−1,1), the distribution function of $\eta_{t}$ is represented by Lemma 6.

**Lemma** **6.**  *If the mutation operator of BSO-II $\vec{z}$ obeys U(−1,1) and $Pb^{\prime }<Pb$, its distribution function $F\left(u\right)=P\left(\eta_{t}\leq u\right)$ of the gain $\eta_{t}$ is*

(40) $$\begin{array}{c} F\left(u\right)=\left\{\begin{array}{cc} 0, & u<0\\ \frac{1}{2}, & u=0\\ \frac{\sqrt{3}}{\sqrt{4\pi nk}}\int_{-\infty }^{u}e^{-\frac{3t^{2}}{4nk^{2}}}t, & u>0 \end{array}\right.. \end{array}$$

The proof of Lemma 6 is based on the same principle as Lemma 4. The proof is given as follows, which is used to support the proof of Theorem 7.

**Proof.**  According to the definition of $\eta_{t},t=0,1,\ldots$,

(1) If $f\left({\vec{y}}^{t}\right)\leq f\left({\vec{x}}^{t}\right),\eta_{t}=0$.
(2) If $f\left({\vec{y}}^{t}\right)>f\left({\vec{x}}^{t}\right),\eta_{t}=k\left[\left(\Delta x_{1}^{t}+\Delta x_{2}^{t}+\ldots +\Delta x_{n}^{t}\right)+\left(z_{1}^{t}+z_{2}^{t}+\ldots +z_{n}^{t}\right)\right]$

Since $z_{i}∼U\left(-1,1\right)$, $\Delta x_{i}∼U\left(-1,1\right)$, and $z_{1},\ldots ,z_{n}$ are independent of each other, $\Delta x_{1},\Delta x_{2},\ldots ,\Delta x_{n}$ are also independent of each other. $f({\vec{z}}^{t})$ obeys $N(0,\frac{2}{3}nk^{2})$ according to the Lindeberg–Levy center limit theorem.

Hence, the $\eta_{t}$ distribution function $F\left(u\right)=P\left(\eta_{t}\leq u\right)$ is

(1) If u<0, F(u)=0.
(2) If u=0, $F\left(u\right)=P\left(\eta_{t}\leq u\right)=P\left(\eta_{t}=0\right)=\frac{1}{2}$.
(3) If u>0, $F\left(u\right)=P\left(\eta_{t}\leq u\right)=\frac{\sqrt{3}}{\sqrt{4\pi n}k}\int_{-\infty }^{u}e^{-\frac{3t^{2}}{4nk^{2}}}t$.

□

**Theorem** **7.**  *If the mutation operator of BSO-II $\vec{z}$ obeys U(−1,1), the upper bound on the expected first hitting time to reach the target fitness value na is derived as*

(41) $$\begin{array}{c} E\left(T_{\varepsilon }|X_{0}\right)\leq 1+\frac{5\sqrt{6\pi n}}{4+\sqrt{2}}\frac{a}{k}-\frac{5\sqrt{6\pi }}{4\sqrt{n}+\sqrt{2n}}\frac{\varepsilon }{k}. \end{array}$$

The proof of this theorem is based on the same principle as Theorem 5. The proof is given as follows.

**Proof.**  According to Lemma 6, we have

(42) $$\begin{array}{c} E\left(X_{t}-X_{t+1}|X_{t},A\right)=E\left(\eta_{t}|X_{t},A\right)=\int_{0}^{+\infty }u\left(\frac{\sqrt{3}}{\sqrt{4\pi n}k}\int_{-\infty }^{u}e^{-\frac{3t^{2}}{4nk^{2}}}t\right)=\frac{k\sqrt{n}}{\sqrt{3\pi }}. \end{array}$$

Suppose that the probability Pb=0.2; according to Theorem 1, the following conclusions can be drawn:

(43) $$\begin{array}{cc} \; & E(X_{t}-X_{t+1}|X_{t})\\ \; & =P\left(\bar{A}\right)E\left(X_{t}-X_{t+1}|X_{t},\bar{A}\right)+P\left(A\right)E\left(X_{t}-X_{t+1}|X_{t},A\right)\\ \; & =\left(1-0.2\right)\times E\left(X_{t}-X_{t+1}|X_{t},\bar{A}\right)+0.2\times E\left(X_{t}-X_{t+1}|X_{t},A\right)\\ \; & =0.8\times \frac{k\sqrt{n}}{\sqrt{6\pi }}+0.2\times \frac{k\sqrt{n}}{\sqrt{3\pi }}=\frac{4k\sqrt{n}+k\sqrt{2n}}{5\sqrt{6\pi }}, \end{array}$$

The algorithm starts from the origin at initialization, ${\vec{x}}^{0}=\left(0,0,\ldots ,0\right)$, i.e., $X_{t}=na$. According to Theorem 1, the upper bound on the expected first hitting time is derived as

(44) $$\begin{array}{cc} \; & E\left(T_{\varepsilon }|X_{0}\right)\leq 1+\int_{\varepsilon }^{na}\frac{5\sqrt{6\pi }}{4\sqrt{n}+\sqrt{2n}}x=1+\frac{5\sqrt{6\pi n}}{4+\sqrt{2}}\frac{a}{k}-\frac{5\sqrt{6\pi }}{4\sqrt{n}+\sqrt{2n}}\frac{\varepsilon }{k}.\;\;\;□ \end{array}$$

Theorem 7 indicates that if the mutation operator of BSO-II $\vec{z}$ obeys U(−1,1), its computational time complexity for the equal coefficient linear function is $E\left(T_{\varepsilon }|X_{0}\right)=O\left(\sqrt{n}\right)$.

### 3.3. Summary

Overall, we summarize the theoretical analysis results of the running-time analysis of the single individual BSO in solving the *n*-dimensional equal coefficient linear function in six different situations. The theoretical analysis results are shown in Table 1. The time complexity of the single individual BSO in these six cases is $O(\sqrt{n})$. However, the coefficients in the display expressions are different.

Table 1 shows the correlation between the expected first hitting time, the dimension *n*, the slope *k*, and the parameter *a*. The upper bounds on the expected first hitting time of BSO-II are lower than those of BSO-I. This means that the performance of BSO-II is better than that of BSO-I in solving the equal coefficient linear function. The disrupting operation in BSO helps to reduce the running time of the algorithm. Moreover, the upper bounds on the expected first hitting time of the algorithm using the standard normal distribution mutation operator are lower than those of the algorithms with the uniform distribution mutation operator. In addition, the upper bounds on the expected first hitting time of the algorithm using the $U\left(-\frac{1}{2},\frac{1}{2}\right)$ mutation operator are approximately two times higher than those of the algorithm using the U(−1,1) mutation operator.

## 4. Experimental Results

In Section 3, we obtain the theoretical analysis results of the expected first hitting time of single individual BSOs through the average gain model. To verify the correctness of the analysis results, numerical experiments are presented in this section.

As the number of samples increases, the arithmetic mean will gradually approach true mathematical expectations based on the Wiener–Khinchine theorem of large numbers [58]. The Wiener–Khinchine theorem of large numbers is introduced as follows.

Suppose that $X_{1},X_{2},\ldots$ is a sequence of independent and identically distributed random variables with $E(X_{i})=\mu$; for any ε>0, the following equation will hold.

(45) $$\begin{array}{c} \lim_{n\to \infty }P\left(\left|\frac{1}{n}\sum_{i=1}^{n}X_{i}-\mu \right|<\varepsilon \right)=1. \end{array}$$

The Wiener–Khinchine theorem of large numbers indicates that if the number of samples is sufficiently large, the mathematical expectations are approximately equal to the mean of samples $X_{1},X_{2},\ldots ,X_{n}$. Therefore, we use the arithmetic mean of the first hitting time of multiple experiments to estimate the actual expected first hitting time.

In this section, the parameters of the proposed approach are set as follows. The fixing error is $\varepsilon =1\times 10^{-8}$, the initial individual is $x^{0}=\left(x_{1}^{0},x_{2}^{0},\ldots ,x_{n}^{0}\right)=\left(0,0,\ldots ,0\right)$, the slope is k=1, and the target fitness parameter is a=10. The problem dimension *n* is set from 10 to 280. BSO-I and BSO-II are conducted on the *n*-dimensional equal coefficient linear function for 300 runs for each dimension. The termination criterion for each experiment is that the error of the optimal solution should be below a predefined threshold ε. Table 2 shows the numerical results of the practical expected first hitting time $\hat{E(T_{\varepsilon }|X_{0})}$ and the theoretical time upper bound, where $\hat{E(T_{\varepsilon }|X_{0})}=\frac{\sum_{i=1}^{300}T_{\varepsilon i}}{300}$, and $T_{\varepsilon i}$ is the first hitting time of the ε-approximation solution at the *i*-th run.

**Figure 1:**
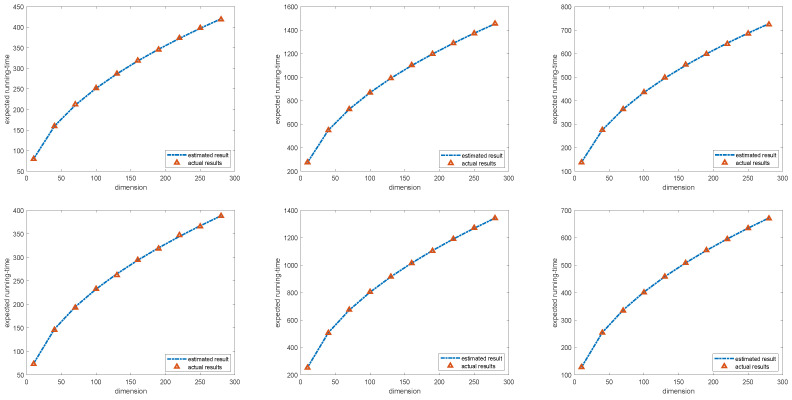
The curve of the expected and the actual running time. The six figures, arranged from top to bottom and left to right, depict the three distributions corresponding to BSO-I and the three distributions corresponding to BSO-II, respectively.

As shown in Table 2, the experimental results strongly fit the theoretical upper bounds, indicating that the error between the theoretical upper bounds and the actual value is within ε. The points larger than the theoretical upper bounds are highlighted in boldface. The arithmetic mean of multiple experiments is used to estimate the expected first hitting time. In the real case, only the arithmetic mean of 300 experiments is used to estimate the expected first hitting time, which allows a certain statistical error. According to the central limit theorem, the results obtained from 300 independent experiments follow a normal distribution. The null hypothesis $H_{0}$ means that the mean value of the expected first hitting time in 300 experiments is less than or equal to the corresponding theoretical upper bounds. The corresponding significance level α is 0.05 with the *T* testing. Moreover, as shown in Figure 1, the actual expected running time is followed with the estimated result based on our proof. All the detailed results are shown in Table 3.

Table 3 provides the numerical results, where *h* represents the hypothetical result, *p* represents the *p*-value of the test, and ci is the confidence interval. As shown in the *T* testing of Table 3, h=0 and p>α. The null hypothesis $H_{0}$ is accepted at the significance level α=0.05. Therefore, the analytic expression of the running time of BSO obtained based on the average gain model can characterize the actual upper bounds of the running time of BSO in these six BSO variants.

## 5. Conclusions

The running time of six BSO variants for the equal coefficient linear function is analyzed in this paper based on the average gain model. The additivity of the normal distribution and the Lindeberg–Levy center limit theorem are applied to deal with the superposition of the normal distribution mutation operator and the uniform distribution mutation operator, respectively. Furthermore, the full expectation formula is utilized to deal with the problem of individual replacement with a certain probability in the disrupting operator. This paper also concludes the upper bounds on the expected first hitting time of the single individual BSO in equal coefficient linear functions.

The analysis results show that the time complexity of BSO-I and BSO-II is $O(\sqrt{n})$ in the equal coefficient linear function. However, their coefficients are different. In the linear function with equal coefficients, the upper bound of the expected first hit time of BSO-II is smaller than that of BSO-I. In addition, the single individual BSO using the standard normally distributed mutation operator expects a lower upper bound on the first hit time than the corresponding algorithm using the uniformly distributed mutation operator. The upper bounds on the expected first hitting time of single individual BSOs with the $U(-\frac{1}{2},\frac{1}{2})$ mutation operator are approximately twice those of BSOs with the U(−1,1) mutation operator.

In our future work, we will analyze the running time of the population-based BSO in the equal coefficient linear function. The running time of population-based BSOs in practical optimization problems is also an important topic. Moreover, it is crucial to extend our research to practical optimization problems that are encountered in real-world applications with complex constraints, non-linear relationships, or high-dimensional spaces. 

## Figures and Tables

**Table 1:** Analysis of the running time of BSO in six different situations.

Algorithm	Mutation Operator $\vec{z}$	Display Expression for $E(T_{\varepsilon }|X_{0})$	Time Complexity T(n)
BSO-I	N(0,1)	$E\left(T_{\varepsilon }|X_{0}\right)\leq 1+\sqrt{2\pi n}\frac{a}{k}-\sqrt{\frac{2\pi }{n}}\frac{\varepsilon }{k}$	$O(\sqrt{n})$
	$U\left(-\frac{1}{2},\frac{1}{2}\right)$	$E\left(T_{\varepsilon }|X_{0}\right)\leq 1+2\sqrt{6\pi n}\frac{a}{k}-\frac{2\sqrt{6\pi }}{\sqrt{n}}\frac{\varepsilon }{k}$	$O(\sqrt{n})$
	U(−1,1)	$E\left(T_{\varepsilon }|X_{0}\right)\leq 1+\sqrt{6\pi n}\frac{a}{k}-\sqrt{\frac{6\pi }{n}}\frac{\varepsilon }{k}$	$O(\sqrt{n})$
BSO-II	N(0,1)	$E\left(T_{\varepsilon }|X_{0}\right)\leq 1+\frac{5\sqrt{2\pi n}}{4+\sqrt{2}}\frac{a}{k}-\frac{5\sqrt{2\pi }}{4\sqrt{n}+\sqrt{2n}}\frac{\varepsilon }{k}$	$O(\sqrt{n})$
	$U\left(-\frac{1}{2},\frac{1}{2}\right)$	$E\left(T_{\varepsilon }|X_{0}\right)\leq 1+\frac{10\sqrt{6\pi n}}{4+\sqrt{2}}\frac{a}{k}-\frac{10\sqrt{6\pi }}{4\sqrt{n}+\sqrt{2n}}\frac{\varepsilon }{k}$	$O(\sqrt{n})$
	U(−1,1)	$E\left(T_{\varepsilon }|X_{0}\right)\leq 1+\frac{5\sqrt{6\pi n}}{4+\sqrt{2}}\frac{a}{k}-\frac{5\sqrt{6\pi }}{4\sqrt{n}+\sqrt{2n}}\frac{\varepsilon }{k}$	$O(\sqrt{n})$

**Table 2:** Comparison of the estimation of the expected first hitting time and the theoretical upper bounds.

Algorithm	$\vec{z}$	*n*	10	40	70	100	130	160	190	220	250	280
BSO-I	N(0,1)	$1+\sqrt{2\pi n}\frac{a}{k}-\sqrt{\frac{2\pi }{n}}\frac{\varepsilon }{k}$	80.27	159.53	210.72	251.66	286.80	318.07	346.51	372.79	397.33	420.44
		$\hat{E(T_{\varepsilon }|X_{0})}$	79.65	**159.57**	**212.51**	**252.09**	**287.14**	316.84	345.61	**373.66**	**398.08**	418.75
	$U\left(-\frac{1}{2},\frac{1}{2}\right)$	$1+2\sqrt{6\pi n}\frac{a}{k}-\frac{2\sqrt{6\pi }}{\sqrt{n}}\frac{\varepsilon }{k}$	275.59	550.17	727.49	869.32	991.04	1099.35	1197.90	1288.93	1373.94	1453.98
		$\hat{E(T_{\varepsilon }|X_{0})}$	**275.81**	548.63	**727.97**	868.67	990.57	**1102.96**	**1198.00**	1288.83	1373.06	**1455.95**
	U(−1,1)	$1+\sqrt{6\pi n}\frac{a}{k}-\sqrt{\frac{6\pi }{n}}\frac{\varepsilon }{k}$	138.29	275.59	364.24	435.16	496.02	550.17	599.45	644.96	687.47	727.49
		$\hat{E(T_{\varepsilon }|X_{0})}$	137.27	275.45	363.95	**435.81**	**498.34**	**553.30**	598.41	641.36	685.46	723.91
BSO-II	N(0,1)	$1+\frac{5\sqrt{2\pi n}}{4+\sqrt{2}}\frac{a}{k}-\frac{5\sqrt{2\pi }}{4\sqrt{n}+\sqrt{2n}}\frac{\varepsilon }{k}$	74.20	147.40	194.68	232.49	264.93	293.81	320.08	344.35	367.01	388.35
		$\hat{E(T_{\varepsilon }|X_{0})}$	72.97	145.55	193.03	**232.77**	262.05	**294.26**	318.51	**347.10**	365.51	387.87
	$U\left(-\frac{1}{2},\frac{1}{2}\right)$	$1+\frac{10\sqrt{6\pi n}}{4+\sqrt{2}}\frac{a}{k}-\frac{10\sqrt{6\pi }}{4\sqrt{n}+\sqrt{2n}}\frac{\varepsilon }{k}$	254.58	508.16	671.91	802.89	915.30	1015.32	1106.33	1190.40	1268.90	1342.82
		$\hat{E(T_{\varepsilon }|X_{0})}$	251.80	505.49	**674.53**	**804.58**	**915.61**	1014.22	1103.21	**1190.82**	**1271.88**	1342.12
	U(−1,1)	$1+\frac{5\sqrt{6\pi n}}{4+\sqrt{2}}\frac{a}{k}-\frac{5\sqrt{6\pi }}{4\sqrt{n}+\sqrt{2n}}\frac{\varepsilon }{k}$	127.79	254.58	336.45	401.95	458.15	508.16	553.67	595.70	634.95	671.91
		$\hat{E(T_{\varepsilon }|X_{0})}$	**128.10**	253.61	334.30	400.20	458.07	507.69	**554.34**	594.77	634.45	669.75

The points larger than the theoretical upper bounds are highlighted in boldface.

**Table 3:** Statistical results of hypothesis testing.

Algorithm	$\vec{z}$	*n*	10	40	70	100	130	160	190	220	250	280
BSO-I	N(0,1)	*h*	0	0	0	0	0	0	0	0	0	0
		*p*	0.80	0.48	0.06	0.38	0.41	0.81	0.72	0.31	0.34	0.83
		ci	78.41	157.86	210.57	249.84	284.76	314.52	343.03	370.82	395.13	415.87
			Inf	Inf	Inf	Inf	Inf	Inf	Inf	Inf	Inf	Inf
	$U\left(-\frac{1}{2},\frac{1}{2}\right)$	*h*	0	0	0	0	0	0	0	0	0	0
		*p*	0.44	0.79	0.42	0.60	0.57	0.10	0.49	0.51	0.61	0.27
		ci	273.43	545.49	724.19	864.51	986.22	1098.22	1193.50	1283.96	1367.75	1450.62
			Inf	Inf	Inf	Inf	Inf	Inf	Inf	Inf	Inf	Inf
	U(−1,1)	*h*	0	0	0	0	0	0	0	0	0	0
		*p*	0.85	0.54	0.57	0.36	0.10	0.07	0.69	0.95	0.81	0.94
		ci	135.64	273.05	361.29	432.88	495.38	549.76	595.05	637.79	681.73	720.20
			Inf	Inf	Inf	Inf	Inf	Inf	Inf	Inf	Inf	Inf
BSO-II	N(0,1)	*h*	0	0	0	0	0	0	0	0	0	0
		*p*	0.95	0.96	0.93	0.42	0.99	0.38	0.83	0.06	0.81	0.61
		ci	71.71	143.86	191.16	230.52	259.89	291.83	315.84	344.26	362.73	385.06
			Inf	Inf	Inf	Inf	Inf	Inf	Inf	Inf	Inf	Inf
	$U\left(-\frac{1}{2},\frac{1}{2}\right)$	*h*	0	0	0	0	0	0	0	0	0	0
		*p*	0.98	0.92	0.12	0.23	0.45	0.65	0.86	0.44	0.18	0.59
		ci	249.47	502.38	670.91	800.79	911.60	1009.53	1098.54	1185.87	1266.44	1337.20
			Inf	Inf	Inf	Inf	Inf	Inf	Inf	Inf	Inf	Inf
	U(−1,1)	*h*	0	0	0	0	0	0	0	0	0	0
		*p*	0.37	0.77	0.92	0.84	0.52	0.60	0.37	0.66	0.59	0.83
		ci	126.53	251.47	331.78	397.34	455.24	504.80	551.07	591.02	630.94	665.97
			Inf	Inf	Inf	Inf	Inf	Inf	Inf	Inf	Inf	Inf

## Data Availability

Data are contained within the article.

## References

[1] Michaloglou A., Tsitsas N.L. (2023). A Brain Storm and Chaotic Accelerated Particle Swarm Optimization Hybridization. Algorithms.

[2] Slowik A., Kwasnicka H. (2017). Nature inspired methods and their industry applications-swarm intelligence algorithms. IEEE Trans. Ind. Inform..

[3] Xue Y., Jiang J., Zhao B., Ma T. (2018). A self-adaptive artificial bee colony algorithm based on global best for global optimization. Soft Comput..

[4] Liu X., Zhan Z., Gao Y., Zhang J., Kwong S., Zhang J. (2019). Coevolutionary Particle Swarm Optimization With Bottleneck Objective Learning Strategy for Many-Objective Optimization. IEEE Trans. Evol. Comput..

[5] Zhao Q., Li C. (2020). Two-Stage Multi-Swarm Particle Swarm Optimizer for Unconstrained and Constrained Global Optimization. IEEE Access.

[6] Yu X., Chen W., Gu T., Yuan H., Zhang H., Zhang J. (2019). ACO-A*: Ant Colony Optimization Plus A* for 3-D Traveling in Environments with Dense Obstacles. IEEE Trans. Evol. Comput..

[7] Lyu Z., Wang Z., Duan D., Lin L., Li J., Yang Y., Chen Y., Li Y. (2020). Tilting Path Optimization of Tilt Quad Rotor in Conversion Process Based on Ant Colony Optimization Algorithm. IEEE Access.

[8] Zhang L., Wang S., Zhang K., Zhang X., Sun Z., Zhang H., Chipecane M.T., Yao J. (2019). Cooperative Artificial Bee Colony Algorithm with Multiple Populations for Interval Multiobjective Optimization Problems. IEEE Trans. Fuzzy Syst..

[9] Kumar D., Mishra K. (2018). Co-variance guided artificial bee colony. Appl. Soft Comput..

[10] Cheng S., Qin Q., Chen J., Shi Y. (2016). Brain storm optimization algorithm: A review. Artif. Intell. Rev..

[11] Cheng S., Sun Y., Chen J., Qin Q., Chu X., Lei X., Shi Y. A comprehensive survey of brain storm optimization algorithms. Proceedings of the 2017 IEEE Congress on Evolutionary Computation (CEC).

[12] Cheng S., Lei X., Hui L., Zhang Y., Shi Y. (2019). Generalized pigeon-inspired optimization algorithms. Sci. China Inf. Sci..

[13] Xiong G., Shi D., Zhang J., Zhang Y. (2018). A binary coded brain storm optimization for fault section diagnosis of power systems. Electr. Power Syst. Res..

[14] Wang Z., He J., Xu Y., Crossley P., Zhang D. (2018). Multi-objective optimisation method of power grid partitioning for wide-area backup protection. IET Gener. Transm. Distrib..

[15] Ogawa S., Mori H. A Hierarchical Scheme for Voltage and Reactive Power Control with Predator-Prey Brain Storm Optimization. Proceedings of the 2019 20th International Conference on Intelligent System Application to Power Systems (ISAP).

[16] Matsumoto K., Fukuyama Y. Voltage and Reactive Power Control by Parallel Modified Brain Storm Optimization. Proceedings of the 2020 International Conference on Artificial Intelligence in Information and Communication (ICAIIC).

[17] Soyinka O.K., Duan H., Tan Y., Shi Y., Niu B. (2016). Optimal Impulsive Thrust Trajectories for Satellite Formation via Improved Brainstorm Optimization. Proceedings of the Advances in Swarm Intelligence.

[18] Li J., Duan H. (2015). Simplified brain storm optimization approach to control parameter optimization in F/A-18 automatic carrier landing system. Aerosp. Sci. Technol..

[19] Zhang C., Xu X., Shi Y., Deng Y., Li C., Duan H. Binocular Pose Estimation for UAV Autonomous Aerial Refueling via Brain Storm Optimization. Proceedings of the 2019 IEEE Congress on Evolutionary Computation (CEC).

[20] Tuba E., Strumberger I., Zivkovic D., Bacanin N., Tuba M. Mobile Robot Path Planning by Improved Brain Storm Optimization Algorithm. Proceedings of the 2018 IEEE Congress on Evolutionary Computation (CEC).

[21] Li G., Zhang D., Shi Y. An Unknown Environment Exploration Strategy for Swarm Robotics Based on Brain Storm Optimization Algorithm. Proceedings of the 2019 IEEE Congress on Evolutionary Computation (CEC).

[22] Aldhafeeri A., Rahmat-Samii Y. (2019). Brain Storm Optimization for Electromagnetic Applications: Continuous and Discrete. IEEE Trans. Antennas Propag..

[23] Sun Y. (2014). A Hybrid Approach by Integrating Brain Storm Optimization Algorithm with Grey Neural Network for Stock Index Forecasting. Abstr. Appl. Anal..

[24] Xiong G., Shi D. (2018). Hybrid biogeography-based optimization with brain storm optimization for non-convex dynamic economic dispatch with valve-point effects. Energy.

[25] Wu Y., Wang X., Xu Y., Fu Y., Cheng S., Shi Y. (2019). Multi-objective Differential-Based Brain Storm Optimization for Environmental Economic Dispatch Problem. Proceedings of the Brain Storm Optimization Algorithms: Concepts, Principles and Applications.

[26] Ma X., Jin Y., Dong Q. (2017). A generalized dynamic fuzzy neural network based on singular spectrum analysis optimized by brain storm optimization for short-term wind speed forecasting. Appl. Soft Comput..

[27] Liang J.J., Wang P., Yue C.T., Yu K., Li Z.H., Qu B. Multi-objective Brainstorm Optimization Algorithm for Sparse Optimization. Proceedings of the 2018 IEEE Congress on Evolutionary Computation (CEC).

[28] Fu Y., Tian G., Fathollahi-Fard A.M., Ahmadi A., Zhang C. (2019). Stochastic multi-objective modelling and optimization of an energy-conscious distributed permutation flow shop scheduling problem with the total tardiness constraint. J. Clean. Prod..

[29] Pourpanah F., Shi Y., Lim C.P., Hao Q., Tan C.J. (2019). Feature selection based on brain storm optimization for data classification. Appl. Soft Comput..

[30] Peng S., Wang H., Yu Q. (2020). Multi-Clusters Adaptive Brain Storm Optimization Algorithm for QoS-Aware Service Composition. IEEE Access.

[31] Zhou Z., Duan H., Shi Y. Convergence analysis of brain storm optimization algorithm. Proceedings of the 2016 IEEE Congress on Evolutionary Computation (CEC).

[32] Qiao Y., Huang Y., Gao Y. (2018). The Global Convergence Analysis of Brain Storm Optimization. NeuroQuantology.

[33] Zhang Y., Huang H., Hongyue W., Hao Z. (2019). Theoretical analysis of the convergence property of a basic pigeon-inspired optimizer in a continuous search space. Sci. China Inf. Sci..

[34] Sudholt D. (2013). A New Method for Lower Bounds on the Running Time of Evolutionary Algorithms. IEEE Trans. Evol. Comput..

[35] He J., Yao X. (2017). Average Drift Analysis and Population Scalability. IEEE Trans. Evol. Comput..

[36] Yu Y., Qian C., Zhou Z.H. (2015). Switch Analysis for Running Time Analysis of Evolutionary Algorithms. IEEE Trans. Evol. Comput..

[37] Li Y., Xiang Z., Ji D. (2019). Wave models and dynamical analysis of evolutionary algorithms. Sci. China Inf. Sci..

[38] Lehre P.K., Witt C., Ahn H.K., Shin C.S. (2014). Concentrated Hitting Times of Randomized Search Heuristics with Variable Drift. Proceedings of the Algorithms and Computation.

[39] Witt C. (2014). Fitness levels with tail bounds for the analysis of randomized search heuristics. Inf. Process. Lett..

[40] Yu Y., Qian C. Running time analysis: Convergence-based analysis reduces to switch analysis. Proceedings of the 2015 IEEE Congress on Evolutionary Computation (CEC).

[41] Huang H., Xu W., Zhang Y., Lin Z., Hao Z. (2014). Runtime analysis for continuous (1 + 1) evolutionary algorithm based on average gain model. Sci. China Inf. Sci..

[42] Huang H., Su J., Zhang Y., Hao Z. (2020). An Experimental Method to Estimate Running Time of Evolutionary Algorithms for Continuous Optimization. IEEE Trans. Evol. Comput..

[43] Zhang Y., Huang H., Hao Z., Hu G. (2016). First hitting time analysis of continuous evolutionary algorithms based on average gain. Clust. Comput..

[44] Yu Y., Zhou Z.H. (2006). A new approach to estimating the expected first hitting time of evolutionary algorithms. Artif. Intell..

[45] Wang Y. (2023). Application of data mining based on swarm intelligence algorithm in financial support of livestock and poultry breeding insurance. Soft Comput..

[46] Zhan Z., Zhang J., Shi Y., Liu H. A modified brain storm optimization. Proceedings of the 2012 IEEE Congress on Evolutionary Computation.

[47] El-Abd M. Brain storm optimization algorithm with re-initialized ideas and adaptive step size. Proceedings of the 2016 IEEE Congress on Evolutionary Computation (CEC).

[48] Shi Y., Tan Y., Shi Y., Chai Y., Wang G. (2011). Brain Storm Optimization Algorithm. Proceedings of the Advances in Swarm Intelligence.

[49] Shi Y. (2011). An Optimization Algorithm Based on Brainstorming Process. Int. J. Swarm Intell. Res..

[50] Yao X., Xu Y. (2006). Recent advances in evolutionary computation. J. Comput. Sci. Technol..

[51] Agapie A., Agapie M., Zbaganu G. (2013). Evolutionary algorithms for continuous-space optimisation. Int. J. Syst. Sci..

[52] He J., Yao X. (2001). Drift analysis and average time complexity of evolutionary algorithms. Artif. Intell..

[53] Hassler U. (2016). Riemann Integrals. Proceedings of the Stochastic Processes and Calculus: An Elementary Introduction with Applications.

[54] Jägersküpper J. (2011). Combining Markov-chain analysis and drift analysis: The (1 + 1) evolutionary algorithm on linear functions reloaded. Algorithmica.

[55] Witt C. (2013). Tight Bounds on the Optimization Time of a Randomized Search Heuristic on Linear Functions. Comb. Probab. Comput..

[56] Hao Z., Huang H., Zhang X., Tu K. A Time Complexity Analysis of ACO for Linear Functions. Proceedings of the Simulated Evolution and Learning, 6th International Conference, SEAL 2006.

[57] Jgersküpper J. (2007). Algorithmic analysis of a basic evolutionary algorithm for continuous optimization. Theor. Comput. Sci..

[58] Feller W. (2008). An Introduction to Probability Theory and Its Applications.

[59] Zhan Z.H., Chen W.N., Lin Y., Gong Y.J., Li Y.L., Zhang J. Parameter investigation in brain storm optimization. Proceedings of the 2013 IEEE Symposium on Swarm Intelligence (SIS).

